# E-health intervention for preventing recurrent ankle sprains: a randomised controlled trial in general practice

**DOI:** 10.3399/BJGP.2022.0465

**Published:** 2023-12-29

**Authors:** Adinda KE Mailuhu, Evert ALM Verhagen, John van Ochten, Patrick JE Bindels, Sita MA Bierma-Zeinstra, Marienke van Middelkoop

**Affiliations:** Department of General Practice, Erasmus MC, University Medical Centre, Rotterdam, the Netherlands.; Department of Health Sciences & EMGO Institute for Health and Care Research, VU University Medical Center, Amsterdam, the Netherlands.; Department of General Practice, Erasmus MC, University Medical Centre, Rotterdam, the Netherlands.; Department of General Practice, Erasmus MC, University Medical Centre, Rotterdam, the Netherlands.; Department of General Practice, Erasmus MC, University Medical Centre, Rotterdam, the Netherlands.; Department of General Practice, Erasmus MC, University Medical Centre, Rotterdam, the Netherlands.

**Keywords:** ankle sprains, e-health, general practice, neuromuscular training, treatment

## Abstract

**Background:**

Ankle sprains are frequent injuries in general practice. However, no effective treatment is available yet.

**Aim:**

To examine the effectiveness of an unsupervised e-health-supported neuromuscular training programme in combination with usual care compared with usual care alone in patients with acute lateral ankle sprains in general practice.

**Design and setting:**

Randomised controlled trial with 1-year follow-up among patients (14–65 years) who visited the GP with an acute lateral ankle sprain within 3 weeks of injury.

**Method:**

The intervention group received, in addition to usual care, an unsupervised e-health-supported neuromuscular training programme and the control group received usual care alone. The primary outcome was self-reported re-sprains during 52 weeks of follow-up. Secondary outcomes were ankle function, pain in rest and during activity, subjective recovery, and return to the same type and level of sport.

**Results:**

In total, 165 participants (mean age 38.3 years and 69 [41.8%] male) were included. No statistically significant difference in the occurrence of a re-sprain were found between the intervention 20.7% (17/82) and control group 24.1% (20/83) (hazard ratio 1.14, 95% confidence interval = 0.59 to 2.21). Also, no statistically significant differences in secondary outcomes were found between groups. The adherence rate to the programme was low (6.1%, 5/82).

**Conclusion:**

The rate of re-sprains was relatively high and an unsupervised e-health-supported neuromuscular training programme does not yield meaningful effects and does not encourage adherence in preventing re-sprains in patients in general practice. More research is necessary to indicate the best treatment modality and way of delivery for these patients.

## Introduction

Acute lateral ankle sprains (LASs) are one of the most common injuries of the musculoskeletal system. The incidence rate in the general population is 2.15 per 1000 person-years in the US, with the highest incidence seen in patients aged between 15 and 24.^1^^,^^2^ In a systematic review about the clinical course of an acute ankle sprain, persistent complaints such as pain, recurrences, swelling, and stiffness are reported in up to 33% of patients after 1 year.^3^ A long-term follow-up study in primary care showed that almost 20% of the patients with an ankle sprain reported complaints after 5 years.^4^ Especially in the first year after a LAS, there is an increased risk of a re-sprain.^3^

Given the relatively high risk of re-sprains, effective treatment of LASs is important to prevent long-term complaints and re-sprains. Acute LASs that require medical treatment are often seen by the GP. The Dutch College of General Practitioners’ guideline for ankle sprains recommends different treatment options, such as Rest, Ice, Compression, Elevation (RICE), bracing, or exercises.^5^ However, not every recommended treatment strategy has been shown to be effective.^6^^,^^7^

An 8-week unsupervised neuromuscular training programme examined in a Dutch trial among athletes was effective with a relative recurrence risk reduction of 35%.^8^ Based on this programme, the ‘Versterk je enkel’ (‘Strengthen your ankle’) app was developed.^9^ As patients in general practice experiencing a LAS are relatively young, an e-health intervention may be a useful and easy treatment.

The current study therefore aimed to examine the effectiveness of an app-based unsupervised neuromuscular training programme, in addition to GP-led usual care, compared with GP-led usual care alone in patients with an acute LAS in general practice, in reducing the number of recurrent LASs.

## Method

### Trial design

The trAPP-study was undertaken according to a previously published protocol.^10^ Briefly, a multicentre, open-labelled randomised controlled trial (RCT) in general practice was undertaken with 1 year follow-up. An unsupervised e-health-supported neuromuscular training programme of 8 weeks in addition to GP-led usual care was compared with GP-led usual care alone.

**Table:** How this fits in

Despite ankle sprains being common in general practice, no treatment modality has proven to be effective. This study showed that an unsupervised e-health-supported neuromuscular train-ing programme has low adherence and is, in its current form, not effective in the prevention of re-sprains in patients in general practice. As the recurrence rate in both study groups is relatively high, there is a need to further explore effective interventions with a focus on the type of intervention and improving adherence to the intervention.

### Participants

Patients with an acute LAS (14–65 years) who visited a GP within the 3 weeks after the injury were eligible for inclusion. Exclusion criteria were a LAS during the previous year, a fracture, or no understanding of the Dutch language. Interested patients were referred to the research team by the GP. Additionally, participants were recruited through social media channels (for example, Facebook, Twitter [now known as X]) and advertisements at sports centres and events. All potential participants were screened for eligibility by the research assistant by telephone and provided written informed consent.

### Randomisation procedure

Participants were randomised by a computer-generated randomisation list (block sizes two, four, and six) with a 1:1 allocation ratio, to receive either the app-based programme, in addition to GP-led usual care (intervention group) or GP-led usual care alone (control group). An independent data manager created the randomisation list and this was concealed for other involved researchers.

### Interventions

The control group only received GP-led usual care. The content of usual care was based on the Dutch Guideline for GPs and best practice.^10^

The intervention group received, in addition to GP-led usual care, an 8-week standardised neuromuscular training programme. The free application ‘Versterk je enkel’ (‘Strengthen your ankle’) guided participants through the programme. Participants were instructed to perform three training sessions per week. Every session consisted of six exercises, which became more difficult in time and were performed in different conditions (with eyes open or shut, with or without a handhold, on an even or uneven surface) (Supplementary Information S1 [in Dutch]).^10^ A personal scheme in the app enabled participants to keep up with their exercises throughout the programme. Participants trained individually and unsupervised.

### Outcomes

Participants completed, after baseline, online questionnaires at 4, 8, 12, 16, 21, 26, 31, 35, 39, 43, 47, and 52 weeks’ follow-up. The baseline questionnaire included questions on demographics, educational level (‘low’, ‘middle’, or ‘high’), comorbidities, paid job (‘yes, <16 hours’, ‘yes, >16 hours’, or ‘no’), sports participation (‘yes’ or ‘no’), minutes of sport participation per week, previous LASs, Ankle Function Score (AFS) (0–100) and Foot and Ankle Disability Index (FADI) (0–100),^11^^,^^12^ pain in rest and during activity (11-point numeric rating scale [NRS]),^13^^,^^14^ and type of treatment by the GP.

All follow-up questionnaires collected information about the previous month and included the occurrence of a recurrent LAS of the index LAS, subjective recovery (measured on a 7-point Likert scale ranging from 1 ‘completely recovered’ to 7 ‘worse than ever’; patients are deemed to be recovered if they rate themselves as ‘completely recovered’ [ = 1] or ‘strongly recovered’ [ = 2] on the Likert scale, whereas those who rate themselves as ‘3, slightly recovered’ to ‘7, worse than ever’ are deemed to be not recovered), AFS (0–100),^11^^,^^12^^,^^15^ and pain in rest and during activity (11-point NRS).^13^^,^^14^ The questionnaires at 4, 8, 12, 26, and 52 weeks additionally collected information on the use of co-interventions and sport participation, that is, the ability to perform the same type and level of sport as before the index LAS. For the control group, the follow-up questionnaire at 52 weeks included a question about whether they had used the app unsupported during follow-up.

The primary outcome was the difference in self-reported recurrent LAS between the intervention and control group during 1-year follow-up. A recurrent LAS was defined as a re-sprain of the index LAS.

Secondary outcomes were differences in ankle function, pain at rest and during activity, subjective recovery, and sport participation (type and level) at 12, 26, and 52 weeks.

### Use of co-intervention

The use of co-interventions was monitored during follow-up by monthly questionnaires and included information on visits to a healthcare professional (for example, GP, sports physician, specialist) and on self-initiated aids and treatment (for example, pain medication, brace, or taping).

### Adherence

The intervention group completed an extra weekly questionnaire on the number of exercises performed. Adherence was determined by the total number of exercises performed per week during the programme and it was defined by completing ≥75% of the total number of exercises in the programme.^16^

### Sample size

A difference of 19% in the incidence of recurrent LASs between the two groups after 1-year follow-up was considered as clinically relevant.^10^ It was estimated that 33% of the participants in the control group would report a recurrent LAS during follow-up.^8^^,^^17^ To detect this difference, with a power of 80% and alpha of 0.05 (two-sided testing), a total of 77 patients per group were needed. Taking a 10% loss to follow-up into account, a total of 172 patients needed to be included.

### Statistical analysis

Differences between the two groups were analysed following the intention-to-treat principle. Cox regression analysis, with adjustment for sex, was performed for comparing recurrence risk between groups and presented as a hazard ratio (HR) with a 95% confidence interval (CI). The first self-reported recurrent LAS was used as the event. Differences between continuous secondary outcomes were examined with linear mixed models using regression techniques for repeated measures and adjusted for age, sex, body mass index (BMI), educational level, and treatment of ankle sprain by GP at baseline. The covariance structure ‘Unstructured’, with the lowest Akaike’s information criterion, was chosen as a data structure in the analyses to model the covariance of repeated measures. The time-point of follow-up were fixed effects. All outcomes measured at the time points before the time-point of interest (including baseline values) were included in the analyses. The intervention effect on continuous secondary outcomes was quantified as the mean difference between trial arms. Mixed-multilevel models, estimated using restricted maximum likelihood, were fitted to analyse repeated measures data as they allow inclusion of participants that provide outcome data in at least one study wave.

Logistic regression models, adjusted for sex, BMI, treatment of ankle sprain by GP at baseline, pain at rest, pain during exercise, FADI, and AFS, were fitted to compare binary outcomes between the trial groups. Differences in subjective recovery and return to the same sport and the same level of sport between the groups were presented as odds ratios (ORs) with their 95% CI. In case of statistically significant differences in the categorical secondary outcomes between the groups, the number needed to treat was calculated (defined as 1 divided by the risk difference between the groups). All analyses were performed in SPSS Statistics (version 25) and *P*-values ≤0.05 were considered statistically significant.

### Not in line with published protocol

In contrast to what is stated in the previously published study protocol, the cost-effectiveness analysis has not yet been performed, and therefore it is not reported in this manuscript.^10^

## Results

**Figure 1.**
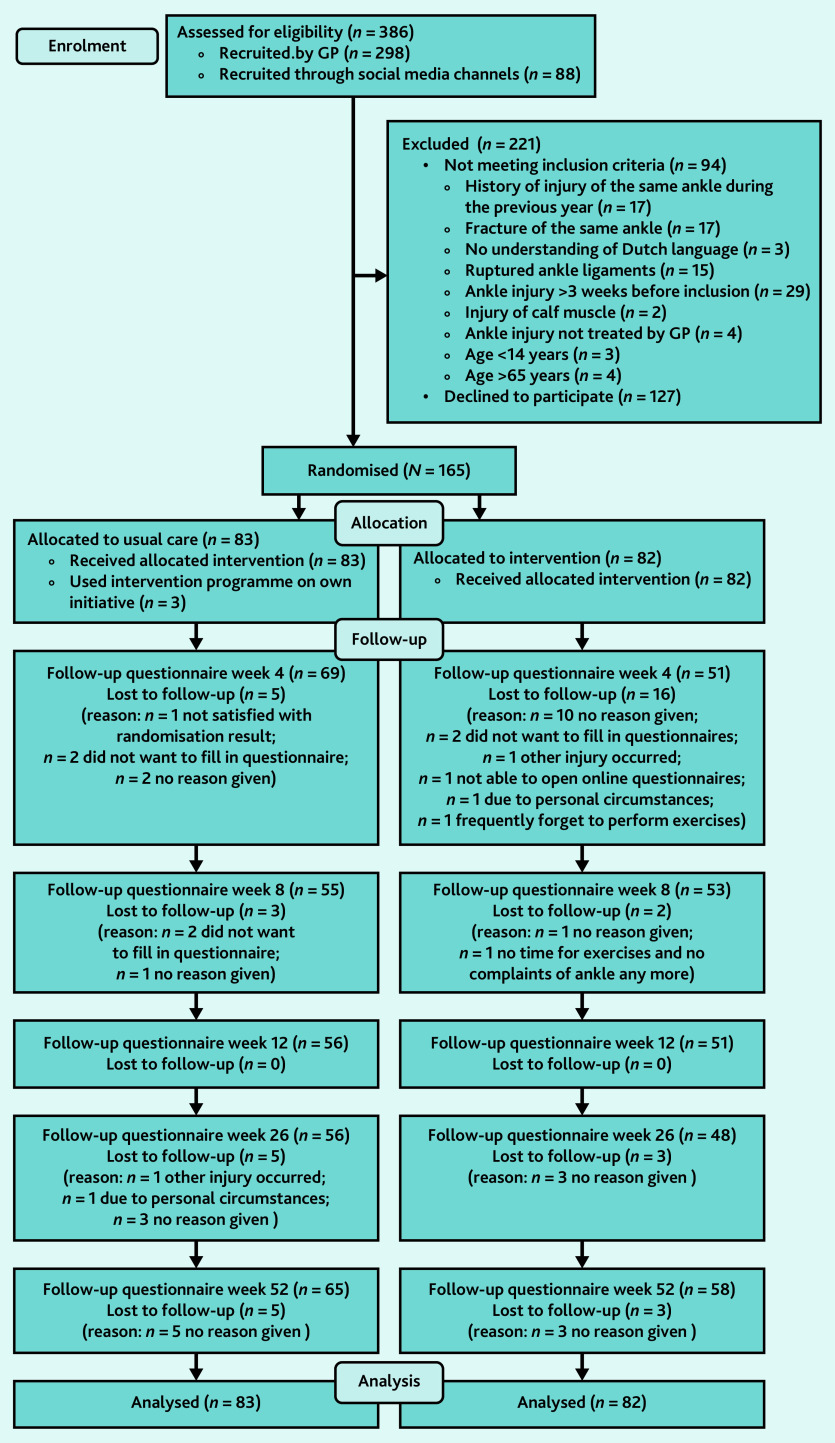
Flowchart of the inclusion of patients in the trAPP-study. Mixed-multilevel model analyses allowed inclusion of all participants who provided outcome data in at least one follow-up measurement.

From November 2014 until January 2018, 386 patients were interested in the study (Figure 1). Of these, 298 (77.2%) patients were registered by their GP and 88 (22.8%) registered through social media channels. After eligibility screening, 165 participants were included and randomised: 83 in the control group and 82 in the intervention group. The mean age was 38.3 years (standard deviation [SD] 14.2) years and 41.8% (69/165) were male (Table 1). Forty-two participants (25.5%) were lost to follow-up: 24 (29.3%) in the intervention and 18 (21.7%) in the control group. Three participants from the control group reported that they used the training programme from the app on their own initiative during follow-up.

**Table 1.** Baseline characteristics of the trAPP-study participants (*n* = 165)

**Characteristic**	**Total population (*n* = 165)**	**Usual care group (*n* = 83)**	**Intervention group (*n* = 82)**
**Age, years, mean (SD)**	38.3 (14.2)	37.1 (15.1)	39.5 (13.3)

**Sex, male, *n* (%)**	69 (41.8)	45 (54.2)	24 (29.3)

**BMI, kg/m^2^, mean (SD)**	24.7 (4.2)	24.2 (4.1)	25.2 (4.3)

**Educational level, *n* (%)**			
Low	31 (18.8)	13 (15.7)	18 (22.0)
Middle	56 (33.9)	30 (36.1)	26 (31.7)
High	78 (47.3)	40 (48.2)	38 (46.3)

**Comorbidities,^a^ *n* (%)**	32 (19.4)	17 (20.5)	15 (18.3)

**Paid job, *n* (%)**			
No	41 (24.8)	21 (25.3)	20 (24.4)
Yes, <16 h	23 (13.9)	12 (14.5)	11 (13.4)
Yes, >16 h	101 (61.2)	50 (60.2)	51 (62.2)

**Sports participation before ankle sprain, *n* (%)**	120 (72.7)	62 (74.7)	58 (70.7)

**Sport participation per week before ankle sprain, min, median (IQR)**	120 (0.0–270.0)	120 (0.0–250.0)	137.5 (0.0–270.8)

**Ankle sprained previously, *n* (%)**	53 (32.1)	26 (31.3)	27 (32.9)

**Pain in rest (NRS 0–10), mean (SD)**	2.2 (2.1)	2.0 (2.0)	2.4 (2.2)

**Pain during exercise (NRS 0–10), mean (SD)**	4.8 (2.6)	4.7 (2.4)	4.9 (2.8)

**AFS (0–100), mean (SD)**	51.9 (20.8)	53.4 (21.3)	50.4 (20.3)

**FADI (0–100), mean (SD)**	70.4 (19.6)	71.8 (19.7)	69.0 (19.5)

**Treatment of ankle sprain by GP,^b^ *n* (%)**			
No treatment	17 (10.3)	11 (13.3)	6 (7.3)
RICE	20 (12.1)	9 (10.8)	11 (13.4)
Analgesics	61 (37.0)	26 (31.3)	35 (42.7)
Bracing or taping	44 (26.7)	23 (27.7)	21 (25.6)
Stimulate physical activity	8 (4.8)	5 (6.0)	3 (3.7)
Physiotherapy	2 (1.2)	1 (1.2)	1 (1.2)
Other	7 (4.2)	2 (2.4)^c^	5 (6.1)^d^

a *Defined as any chronic disease (for example, cardiovascular diseases or musculoskeletal disorders) reported by the participant.*

b *Data missing for 6 participants.*

c *Other treatment of ankle sprain by GP: compression stocking (n = 1); crutches (n = 1).*

d *Other treatment of ankle sprain by GP: compression stocking (n = 3); crutches (n = 1); cast (n = 1). AFS = Ankle Function Score. BMI = body mass index. FADI = Foot and Ankle Disability Index. IQR = interquartile range. NRS = numeric rating score. RICE = Rest, Ice, Compression, and Elevation. SD = standard deviation.*

### Primary outcome

Overall, 22.4% (37/165) of the participants reported one or more re-sprains during follow-up: 24.1% (20/83) in the control group and 20.7% (17/82) in the intervention group. A total of 51 re-sprains were reported by these 37 participants (26 in the intervention and 25 in the control group). There was no statistically significant difference in the occurrence of re-sprains between the two groups (HR 1.14, 95% CI = 0.59 to 2.21, adjusted for sex; unadjusted HR 1.02).

### Secondary outcomes

No differences were observed in any of the secondary outcome measures between the two groups at 12, 26, and 52 weeks (Table 2).

**Table 2.** Secondary study outcomes during 1-year follow-up

**Number of weeks after baseline**	**Usual care group (*n* = 83)**	**Intervention group (*n* = 82)**	**Mean difference (95% CI)^a^**	**OR (95% CI)^b^**
**AFS (0–100), mean (SE)**				
12	68.80 (2.75)	67.55 (2.60)	−1.25 (−5.48 to 2.98)	—
26	75.10 (2.33)	74.31 (2.28)	−0.79 (−4.41 to 2.82)	—
52	77.37 (1.96)	77.04 (1.93)	−0.33 (−3.66 to 3.00)	—

**Pain at rest (NRS 0–10), mean (SE)**				
12	1.84 (0.25)	1.93 (0.25)	0.09 (−0.30 to 0.49)	—
26	1.07 (0.14)	1.23 (0.14)	0.16 (−0.12 to 0.45)	—
52	0.98 (0.10)	1.10 (0.10)	0.12 (−0.10 to 0.34)	—

**Pain during activity (NRS 0–10), mean (SE)**				
12	3.47 (0.38)	3.78 (0.37)	0.31 (−0.26 to 0.88)	—
26	2.66 (0.30)	2.88 (0.29)	0.22 (−0.25 to 0.69)	—
52	2.08 (0.22)	2.21 (0.22)	0.14 (−0.24 to 0.51)	—

**Subjective recovery,^c^ *n* (%)^d^**				
12	52/56 (92.9)	45/49 (91.8)	—	1.81 (0.81 to 4.06)
26	51/55 (92.7)	46/48 (95.8)	—	1.42 (0.66 to 3.07)
52	64/65 (98.5)	56/58 (96.6)	—	1.12 (0.55 to 2.27)

**Return to same type of sport, *n* (%)**				
12	35/40 (87.5)	24/30 (80.0)	—	1.16 (0.44 to 3.10)
26	38/42 (90.5)	27/33 (81.8)	—	1.10 (0.48 to 2.54)
52	40/43 (93.0)	39/42 (92.9)	—	0.95 (0.43 to 2.14)

**Return to sport at same level, *n* (%)**				
12	25/40 (62.5)	19/30 (63.3)	—	0.76 (0.32 to 1.80)
26	30/42 (71.4)	24/33 (72.7)	—	0.55 (0.26 to 1.19)
52	36/43 (83.7)	37/42 (88.1)	—	0.60 (0.30 to 1.21)

a *Adjusted for age, sex, BMI, educational level, and treatment of ankle sprain by GP at baseline (for example, other);*

b *Adjusted for sex, BMI, treatment of ankle sprain by GP at baseline (for example, other), pain in rest, pain during exercise, FADI, and AFS.*

c *Measured on a 7-point Likert scale ranging from 1 ‘completely recovered’ to 7 ‘worse than ever’ and dichotomised into ‘recovered’ (that is, ‘1, completely recovered’ or ‘2, strongly recovered’) and ‘not recovered’ (that is, ‘3, slightly recovered’ to ‘7, worse than ever’).*

d *Reference category is ‘not recovered’. AFS = Ankle Function Score. BMI = body mass index. CI = confidence interval. FADI = Foot and Ankle Disability Index. NRS = numeric rating scale. OR = odds ratio. SE = standard error.*

### Co-interventions during follow-up

During the first 12 weeks’ follow-up, the GP and the paramedical healthcare professional were the most visited professionals in both the intervention (27.1% GPs and 27.1% paramedical healthcare professionals) and the control group (29.2% GPs and 27.8% paramedical healthcare professionals) (Table 3). Bracing or taping was self-initiated by 30.6% of the control group and by 32.2% of the intervention group. At 26 and 52 weeks’ follow-up, only a limited number of co-interventions was reported, with no differences between groups.

**Table 3.** Reported co-interventions during follow-up

**Co-intervention**	**Up to 12 weeks**		**At 26 and 52 weeks^a^**	
	**Usual care (*n* = 83)**	**Intervention (*n* = 82)**	**Usual care (*n* = 83)**	**Intervention (*n* = 82)**
**Patients who visited a healthcare practitioner, *n* (%)**				
GP	21/72 (29.2)	16/59 (27.1)	1/67 (1.5)	1/60 (1.7)
Sports physician	0/72 (0.0)	1/59 (1.7)	0/67 (0.0)	1/60 (1.7)
Specialist	1/72 (1.4)	5/59 (8.5)	0/67 (0.0)	0/60 (0.0)
Paramedical^b^	20/72 (27.8)	16/59 (27.1)	4/67 (6.0)	4/60 (6.7)
Other healthcare practitioner	0/72 (0.0)	2/59 (3.4)^c^	0/67 (0.0)	0/60 (0.0)

**Self-initiated aids and treatment, *n* (%)**				
Pain medication	12/72 (16.7)	16/59 (27.1)	2/67 (3.0)	3/60 (5.0)
Brace and/or taping	22/72 (30.6)	19/59 (32.2)	9/67 (13.4)	4/60 (6.7)
Exercises	5/72 (6.9)	2/59 (3.4)	2/67 (3.0)	2/60 (3.3)
Other self-initiated aids and treatment	2/72 (2.8)^d^	6/59 (10.2)^e^	0/67 (0.0)	0/60 (0.0)

a *Reported co-interventions by participants in the previous month reported at either 26 and 52 weeks’ follow-up.*

b *Paramedical co-intervention included a physiotherapist, manual therapist, or exercise therapist.*

c *Other healthcare practitioner: chiropractor (*n *= 1), emergency room (*n *= 1).*

d *Other self-initiated aids and treatment: working boot (*n *= 1), cooling ointment (*n *= 1).*

e *Other self-initiated aids and treatment: massage (*n *= 2), compressive stocking (*n *= 1), crutches (*n *= 3).*

### Adherence

The number of responses to the specific intervention questionnaires ranged from 74.4% (61/82; week 2) to 51.2% (42/82; weeks 4 and 5). Twenty participants (20/82; 24.4%) were adherent with the intervention programme (that is, performed ≥75% of total prescribed exercises) and only five participants (5/82; 6.1%) were completely adherent (that is, three sets of six exercises per week over 8 weeks).

## Discussion

### Summary

In this trial, the effectiveness of an unsupervised e-health-supported training programme, in addition to GP-led usual care, was examined among 165 participants with a LAS in general practice. In contrast with Hupperets *et al* in 2009, who found a significant risk reduction in the occurrence of re-sprains among athletes performing the programme,^8^ the current study did not find an effect on the recurrence rate, nor were any differences found between the two study groups in any of the secondary outcomes after 1-year follow-up.

### Strengths and limitations

The current study is a high-quality pragmatic RCT evaluating an e-health intervention in addition to usual care about the recurrence rate of LASs in general practice. Nevertheless, some limitations need to be addressed. First, the number of included participants was lower than anticipated and the loss to follow-up was 25.5%, which was higher than expected and therefore had an impact on the power of the analyses.^10^ Second, the usual care provided by the GP at baseline consisted of different strategies. The participating GPs could apply their preferred type of usual care, meaning that there were various types of treatment received at baseline. Nevertheless, no differences were observed in the applied usual care at baseline between the two groups, and therefore this does not seem to have had any impact on the findings.

### Comparison with existing literature

To the authors’ knowledge, this is the first RCT to evaluate an unsupervised e-health-supported training programme for patients with an acute LAS in general practice. Rehabilitation programmes for ankle injuries, such as proprioceptive training programmes, have been studied previously in both general and sport populations,^8^^,^^18^^–^^21^ and recurrence risk reductions from 10% to 60% have been reported.^6^^,^^20^^–^^22^ Although the current study did not find a difference in recurrence rate between study groups, the recurrence rate found in the current study falls within the range reported in literature (3%–30%).^20^ Several factors might be responsible for the difference in study outcomes between the current study and those reported in literature.

One of the reasons that the current study found no effect of the training programme could be the study population. Hupperets *et al* found a positive effect among athletes.^8^ The recurrence risk is higher among athletic populations and may therefore increase the likelihood of finding an effect among these populations. Moreover, the willingness to rehabilitate after an ankle injury may be higher in athletes than in the general population. This is reflected in the higher adherence rate in Hupperets *et al* (23%)^8^ compared with the current study (6.1%). In agreement with the current study findings, van Rijn *et al*, in 2007, also found no difference in the occurrence of re-sprains between a supervised exercise intervention programme and a usual care group after 1-year follow-up in a comparable population from general practice.^17^ Although, because of differences in type of interventions, it remains difficult to compare study outcomes. However, it indicates that intervention effects may differ between athletic and general populations, perhaps because of motivation and consequently higher adherence rates in athletes.

The low adherence rate may have been affected by the fact that there was no contact with the research team about the performance and progress of the intervention programme. Inherent to the adherence, the unsupervised component of the programme could be a limitation. The absence of supervision for motivation, and especially quality control of exercises, could be another reason for the low adherence and contribute to the ineffectiveness of the intervention. This is strengthened by the fact that previous studies have shown that supervised rehabilitation, compared with home exercises, seems to be more effective in reducing pain, subjective instability, and function after an ankle sprain.^23^^,^^24^ Although, as results in the literature are conflicting in general populations, it remains questionable whether a supervised intervention will be more effective in preventing re-sprains in general practice.^17^ Moreover, as the app did not seem to encourage adherence, the true efficacy of the intervention programme remains unclear.

### Implications for research and practice

An unsupervised e-health supported neuromuscular training programme, in addition to GP-led usual care, in its current form is not effective and does not encourage adherence in the prevention of a recurrent sprain during 1 year of follow-up in patients with an acute LAS in general practice. More research is needed to determine the best treatment modality and way of delivery for this group of patients.

## Data

The data that support the findings of this study are available from the corresponding author on reasonable request.
